# A Randomized Controlled Trial Comparing Loss versus Gain Incentives to Improve Adherence to an Obesity Treatment Intervention in Adolescents

**DOI:** 10.3390/nu16193363

**Published:** 2024-10-03

**Authors:** Robert M. Siegel, Christopher Kist, Shelley Kirk, Roohi Kharofa, Kristin Stackpole, Amanda Sammons, Linda Dynan, Meghan E. McGrady, JangDong Seo, Elaine Urbina, Nadine A. Kasparian

**Affiliations:** 1 The Heart Institute, Cincinnati Children’s Hospital, Cincinnati, OH 45229, USA; christopher.kist@cchmc.org (C.K.); shelley.kirk@cchmc.org (S.K.); roohi.kharofa@cchmc.org (R.K.); kristin.stackpole@cchmc.org (K.S.); amanda.sammons@cchmc.org (A.S.); jangdong.seo@cchmc.org (J.S.); elaine.urbina@cchmc.org (E.U.); nadine.kasparian@cchmc.org (N.A.K.); 2 Department of Pediatrics, College of Medicine, University of Cincinnati, Cincinnati, OH 45267, USA; meghan.mcgrady@cchmc.org; 3 James M. Anderson Center for Health Systems Excellence, Cincinnati Children’s Hospital, Cincinnati, OH 45229, USA; dynanl@nku.edu; 4 Department of Accounting, Economics, and Finance, Northern Kentucky University, Highland Heights, KY 41099, USA; 5 Division of Behavioral Pediatrics, Medicine & Clinical Psychology, Cincinnati Children’s Hospital, Cincinnati, OH 45229, USA

**Keywords:** childhood, obesity, incentives, gain, loss, behavioral economics, cost-effectiveness

## Abstract

Background/Objectives: Adherence to pediatric obesity treatment can be challenging. Monetary incentives improve adherence to lifestyle interventions, with incentives framed as loss often more effective than those framed as gain. The objectives of this study were to determine if monetary incentives in the form of gift cards would improve adherence to an obesity treatment intervention and whether framing the incentive as either loss or gain affected adherence. Methods: Sixty adolescents with obesity (body mass index of ≥95th percentile for age and sex) were recruited from our pediatric obesity treatment program. They were randomized into one of three groups and given a monthly adherence score (AS) of up to 100 points. These points were based on completing a medical visit, reporting on diet intake, and measuring daily steps on a wearable tracker. The Gain Group (GG), N = 20, started each month with USD 0 in a virtual account and increased their monetary reward up to USD 100 depending on AS. The Loss Group (LG), N = 21, began each month with USD 100 in their virtual account, which decreased based on adherence. The Control Group (CG), N = 19, received USD 10 monthly. Results: Adherence was highest in the GG, with 66.0 points, compared to the LG, with 54.9 points, and CG, with 40.6 points, with *p* < 0.01. The GG had greater adherence to their step goal (14.6) and dietary reporting (18.7) compared to the LG (10.0 and 13.9) and the CG (3.9 and 8.1), *p* < 0.005. Conclusions: Gain-framed incentives are superior to loss-framed ones in improving adherence to pediatric obesity treatments.

## 1. Introduction

Childhood obesity is a major public health issue, with about 40% of adolescents youths being overweight or obese in the United States [1,2]. The obesity epidemic disproportionately affects Hispanic and non-Hispanic black children, yielding increased comorbidities in these youth [1,3]. Pediatric obesity treatment programs with a family-centered approach have proved to be effective in lowering body mass index (BMI) in youth with obesity [4,5,6]. Indeed, the American Academy of Pediatrics (AAP) recommendations for obesity treatment include parental involvement, such as monitoring, limit setting, barrier reduction, managing family conflict, and modifying the home environment [7]. However, a range of barriers to care exist, such as cost, patient disengagement, which increases in adolescence, and lack of family support [8,9,10,11,12]. Many of these barriers, such as the built environment and family time constraints, are exacerbated in minority and low-income communities [8].

Attrition rates for pediatric obesity programs are high (27 to 73%), with minorities and adolescents at the highest risk of attrition [13,14]. Further, even without formal attrition, adherence to regimes tends to wane over time, reducing their effectiveness [15,16]. Rapoff’s model of adherence to pediatric medical regimes identifies three categories of factors associated with adherence: patient/family factors (e.g., demographics and knowledge), regimen factors (e.g., cost and complexity), and disease factors (e.g., duration and perceived severity) [17]. Some indicators for lack of adherence or eventual attrition linked to this framework are travel distance, higher BMI at the start, greater number of obesity-related comorbidities, lack of child engagement, insurance coverage, and parental perception that treatment is not meeting expectations [11,12,18,19]. This study seeks to address a potential source of modification to child motivation.

As indicated, family-centered approaches to obesity treatment face barriers to success due to a lack of child motivation [19]. Strategies to improve engagement for patients and families attending obesity treatment programs could lead to better outcomes [20]. One strategy favored by parents is a regime that incentivizes behavioral change (e.g., physical activity increases) rather than measured outcomes (e.g., BMI change) [21]. Financial incentives are a potential extrinsic motivator to achieve such behavior modifications. The objective of the financial incentive is to keep the participant sufficiently engaged for long enough to observe a delayed or uncertain outcome [19]. Although rewards and financial incentives raise concerns with parents that such incentives might be short-lived motivators and diminish their children’s intrinsic motivation to make healthy lifestyle choices, incentives in the form of money or other valued rewards have been used in both adult and pediatric settings to improve adherence to medical regimens and encourage healthier choices [15,21,22,23,24]. To our knowledge, monetary incentives have not been tested in pediatric obesity treatment, with the exception of a randomized clinical trial of severely obese adolescents treated with meal replacement therapy with and without a financial incentive. Financial incentives with therapy versus therapy alone improved outcomes, which were measured as reductions in BMI and total body fat without increased unhealthy weight control behaviors [25].

Incentives framed as avoiding a potential loss rather than obtaining a potential gain capitalize on the powerful force of loss aversion and seem to be more effective than gain incentives in promoting smoking cessation and physical activity in some studies [26,27,28]. Still, other studies suggest the opposite, with incentives framed as a gain to be superior to loss-framed incentives [29,30]. Much like a survey of 304 parents of obese children who preferred financial incentives that targeted both parent and child and gain-framed rather than loss-framed payments, in a previous survey of 108 youths in our obesity treatment program and their guardians, we reported that the majority predicted that gain-framed incentives would be superior [19,31].

While both gain- and loss-framed incentives have been tested in adolescents, there is no information on which, if any, would be more effective in a pediatric obesity intervention [22]. The immediate objective of this study was to test whether incorporating monetary incentives into an obesity treatment intervention improves engagement and adherence and to specifically examine whether incentives in the form of avoiding a loss or receiving a gain is the superior strategy. Our second objective was to determine which of the two strategies is more cost-effective. We chose a randomized controlled trial design given the generally accepted superiority of the technique when evaluating interventions [32,33].

## 2. Materials and Methods

Sixty youths with obesity (BMI ≥ 95th percentile for age and sex) aged 13 to 18 years who attended the Center for Better Health and Nutrition (CBHN) pediatric obesity treatment program were recruited between 22 July 2022 and 11 July 2023 to participate in this prospective randomized controlled trial. Patients were randomized in equal numbers to one of three incentive groups: the Control Group (CG), Loss Group (LG), and Gain Group (GG). Participants were either new to the program or had not participated in the program for at least 9 months. Study patients participated in the CBHN/Healthworks!, a family-centered intervention in which patients and guardians see a pediatrician, dietitian, and exercise physiologist. The intervention includes monthly visits, exercise classes, educational videos, and customized care plans. Participants in each group were given a Fitbit Inspire 2 wearable tracker (Fitbit, San Francisco, CA, USA) and Fitbit Aria Scale (Fitbit, San Francisco, CA, USA). The tracker gave an objective measure of steps per day, and the scale was applied if weight checks could not be conducted in CBHN offices secondary to possible COVID-19 restrictions; however, this never became an issue during the study period. 

Adherence to the program (Table 1) was measured in points based on steps per day, responding to a daily dietary survey, and completing monthly clinic visits. While the responses to the dietary study were self-reported, adherence points were based only on completing the surveys, not the responses. Although not included in the adherence score, skin carotenoid levels were measured with a VEGGIE METER^®^ (Longevity Link Corp., Salt Lake City, UT, USA), a device that objectively measures an index of fruit and vegetable intake based on skin carotenoid levels [34,35]. At the time of study entry, participants and their guardians viewed a video explaining the study expectations and how the incentive was determined for their specific group. The video had Spanish subtitles for Spanish-speaking families.

### 2.1. The Groups

Control Group (CG): This group had all the standard program features with an in-clinic visit routine (0, 3 and 6 mos.) of the CBHN obesity treatment program. They were given Fitbit wearable devices to measure steps and adherence to meeting the goal of 10,000 steps per day. Participants had the option of monthly physician, dietitian, and exercise Telehealth visits during months when there were no live clinic visits. Both parents/guardians and participants attended in-person clinic visits at 0, 3, and 6 months. Parental attendance was optional at the 1-, 2-, 4-, and 5-month Telehealth visits. While parents were encouraged to support their children’s adherence, reporting and wearing the tracker were the study participants’ responsibility. Patients received daily text messages with a link to complete the dietary survey of four questions outlined in Table 1. While the adherence index was calculated for patients in this group, the only monetary incentive they received was USD ten per month via a “Clin-Card” gift card. Laboratory studies and anthropometric data (Table 2) were collected at entry and study completion. Laboratory studies included High-Density Lipoprotein (HDL), Low-Density Lipoprotein (LDL), Total Cholesterol, and Triglyceride, Hemoglobin A1C percent (HgbA1C%), Alanine Aminotransferase (ALT), and Aspartate Aminotransferase (AST) levels were obtained within 4 weeks before or after the first and last study visit. Patient/family demographics, such as race, ethnicity, insurance type, and family income, were obtained via self-report entered on a study form. 

Gain Group (GG): This group had all the clinical and reporting features of the CG. In addition, this group received an incentive based on the monthly calculation of the adherence index outlined in Table 1, with every point worth USD one. The patient and guardian received a report monthly describing adherence points and the incentive earned in their Clin-Card “account”. The maximum incentive was USD 100, and the minimum was USD 10 total. The patients had a virtual account, and the value of the incentives was USD 0 at the beginning of the month and increased up to USD 100 based on the participant’s adherence score. 

Loss Group (LG): This group had all the features of the GG except for how the incentive was presented. Patients, like those in the GG, had monthly reports on their adherence and incentive. Their virtual account, however, had a value of USD 100 added at the beginning of the month and lost value if adherence metrics were not met to a potential minimum of USD 10. 

### 2.2. Inclusion Criteria

Subjects who met all of the following criteria were eligible for the study:

1. Age 13–18 years old;
2. BMI ≥ 95th percentile for age and sex;
3. The family must understand English or Spanish;
4. The family must have access to a smartphone;
5. The patient must not have participated in an obesity treatment program within the past 9 months

### 2.3. Exclusion Criteria

Subjects who did not meet any of the above criteria, had a sibling in the study, or could not understand the study requirements as deemed by the examining physician were excluded. As the change in weight status was not a primary endpoint in the study, the patient being on a medication known for weight gain or loss was not a reason for exclusion. 

### 2.4. Data Management

All of the data were recorded on study-specific case report forms (CRFs) in either paper-based or electronic formats. After consent, demographic and medical history were collected from the patient’s electronic medical record and entered into the CRF. Other data were collected directly from the patient via survey, data recorded by their Fitbit tracker, blood draw, and physical examinations. The data obtained included the following: insurance status, race/ethnicity, age, sex, blood pressure, weight, height, percent body fat, skeletal muscle mass, fat mass, veggie meter score, laboratory values, medications, BMI, and percent of 95th percentile BMI for age and sex (%BMIp95), determined as per the criteria of the National Center of Health Statistics [36], daily steps (via Fitbit Tracker), and daily food log, via a link to Redcap, sent via a text message. The food logs were carried out the following day to ensure all of the data were entered. The questions were as follows:

1. Did you eat breakfast yesterday? y/n
  (meal eaten before 10:30 a.m.)
2. How many fruits or vegetables were eaten yesterday? 0 to >10 (defined in a separate handout)
3. How many sugary drinks did you drink yesterday? 0 to >10 (defined in a separate handout)
4. Did you eat after 7 p.m. yesterday? y/n

Study data were stored and processed using a RedCap database. Data were managed by an experienced team of clinical data specialists and coordinators using the Data Management Center’s (DMC) standard operating procedures and guidelines. Data were indexed using a study-specific subject number; the key that linked this number to identifying information was stored in a separate, secure location. 

### 2.5. Statistical and Sensitivity Analysis

Means and standard deviations (SDs) were calculated for continuous variables and counts and percentages for categorical variables. Categorical variables were compared using Chi-square or Fisher’s exact tests, dependent on the number of observations. The Wilcoxon signed-rank test was used to test for differences in the continuous variables between the Loss, Gain, and Control groups. The Kruskal–Wallis rank sum test was used to test for differences between the continuous variables of the three groups as it is the preferred test for a 3-way comparison when data are not normally distributed. The Wilcoxon signed-rank test was used to investigate the changes from baseline to study completion (6 months) in %BMIp95, systolic BP, diastolic BP, veggie meter, HDL, LDL, HgA1C%, cholesterol, triglycerides, AST, and ALT. The Wilcoxon signed-rank test was chosen as the data did not meet the condition of normality. Linear mixed-effects regression was used to test for associations of characteristics with adherence with the study group as the main independent variable. Analyses were conducted using R 4.4.0 statistical software (R Core Team, 2024, Vienna, Austria).

For the economic evaluation, a decision tree for the study was created using TreeAge Pro Version 2023 software (Copyright 1988–2023 TreeAge Software, LLC, Williamstown, MA, USA). The provider costs of visits 2, 4, and 5 were bundled together, as the probability of each of the visits was assumed to be equal for all branches in the analysis. A cost-effective analysis was carried out using the actual study equipment costs (incentives, Fitbit, and scale) and values of physician, dietitian, and exercise visits estimated via posted Medicare reimbursements. Three-way sensitivity analyses were performed, varying the probability of adherence in the CG, LG, and GG by 10% higher and lower than the values observed during the trial.

### 2.6. Sample Size Calculation 

Prior to the study, we calculated the sample size required to power this study for a significant difference in the primary outcome of total adherence points. This study was not intended, a priori, to be of power for secondary outcomes. We assessed the proportion of patients needed in each study arm completing the 3- and 6-month study visits to estimate the mean and SD in the adherence score to help with the design of a larger, confirmatory trial if needed. A sample size of N = 20 per study arm produced a two-sided exact 95% CI (i.e., a margin of error) with a width of (0.51; 0.91) when the sample proportion is 0.75. The margin of error has a width of (0.28; 0.73) when the sample proportion is 0.50. A sample size of N = 12 participants retained at the 6-month visit produces a two-sided 95% CI with a distance from the mean to the limits (half-width of the 95% CI) for the mean that is equal to 6.4 when the estimated SD = 10. The mean to the limit is 12.7 when the SD = 20. The half-width for the 95% CI for the SD is 9.9 and 19.8. Thus, enrolling N = 20 per arm was determined to inform the sample size calculations required to design a larger, rigorously powered study if needed. 

### 2.7. Institutional Approval and Consent

This study was conducted in accordance with the Declaration of Helsinki and approved by the Cincinnati Children’s Institutional Review Board, protocol 2022–0012. For those under 18 years of age, written informed consent was obtained from a guardian of each participant, and the participant gave written assent if they were between the ages of 13 to 17, according to institutional standards. For participants 18 years of age, written informed consent was obtained. 

## 3. Results

### 3.1. Group Comparisons

The demographics and baseline characteristics of the 60 adolescents recruited for the study are summarized in Table 2. The average participant age was 15.2 years old; 62% were female; 46% had Medicaid insurance; 67% were white; 25% were black; and 8.3% were Hispanic. All of the baseline characteristics were similar in the groups, except the veggie meter score, which was highest at entry in the GG. 

**Figure 1:**
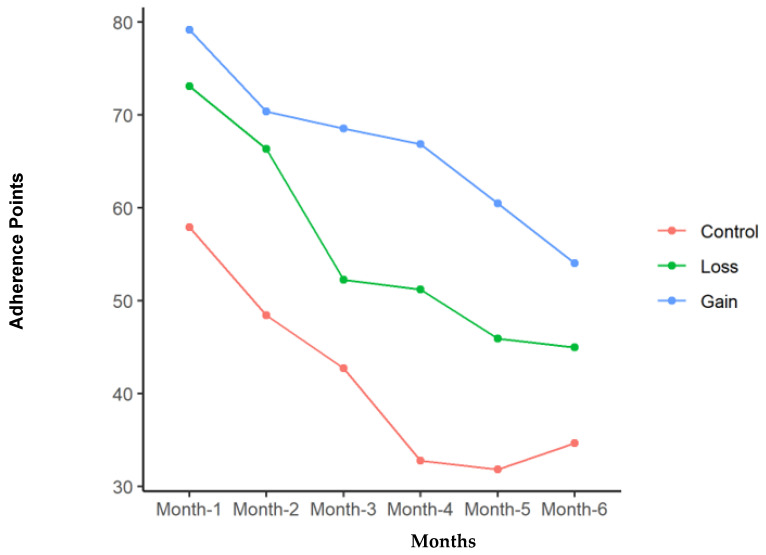
Total adherence points for each of the groups with time in months of study participation.

**Figure 2:**
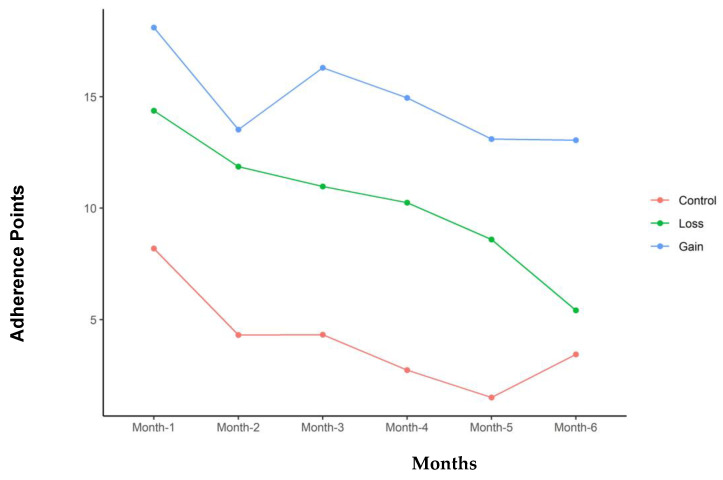
Exercise adherence points for each of the groups with time in months of study participation.

**Figure 3:**
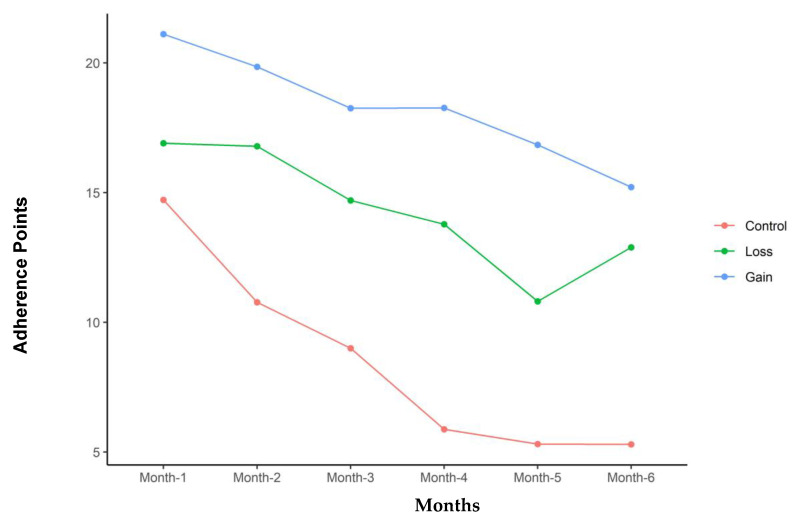
Diet log reporting adherence points for each group with time in months of study participation.

**Figure 4:**
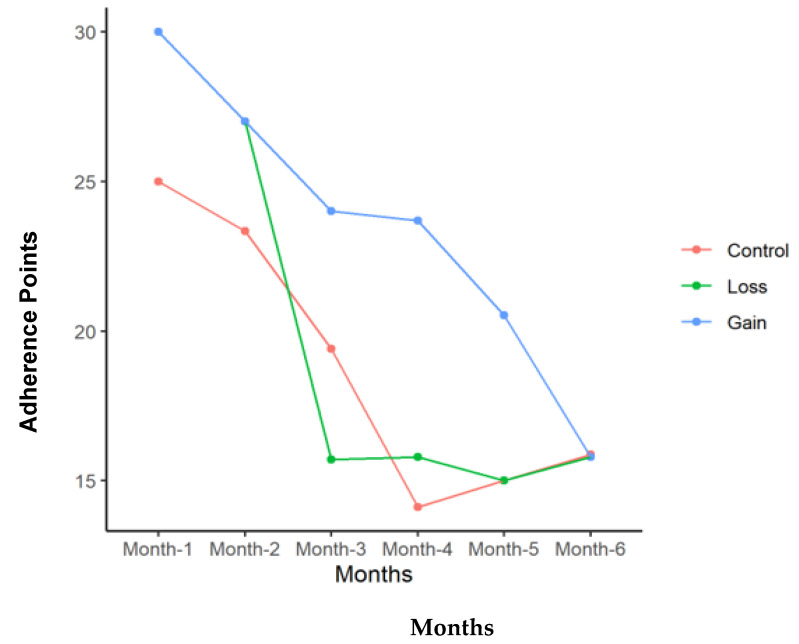
Clinic visit adherence points adherence points for each group with time in months of study participation.

Table 3 summarizes the adherence results of the three groups. The GG overall had the greatest adherence of 66.6 points per month compared to 55.6 points for the LG and 41.4 points for the CG (*p* = 0.009). The GG also had significantly higher adherence to exercise and dietary guidelines. The three groups were more similar in clinic visit adherence, with no significant differences between them. Table 4 addresses Fitbit use during the study and the recorded steps per day. The GG was significantly more likely to wear the tracker; however, the steps per day were not significantly different between the groups when the device was worn. Adherence decreased with time in all three groups. Figure 1, Figure 2, Figure 3 and Figure 4 illustrate how adherence decreased over time for all of the primary outcomes.

**Figure 5:**
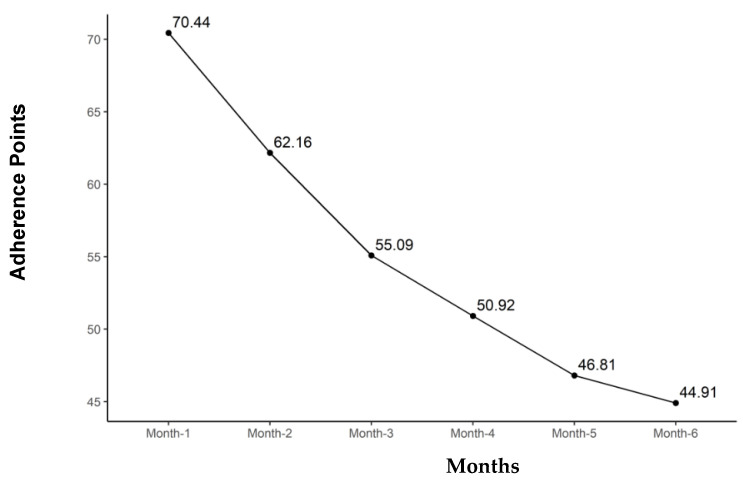
Total adherence points for all groups combined with time in months of study participation.

Figure 5 illustrates the total points for all the groups combined over the study period. Total adherence points declined overall and within all groups over the 6 months (*p* < 0.001), with the mean total adherence for all groups combined dropping from 70.4 ± 24.5 points to 44.9 ± 29.3 points.

Pre- and post-mean values of the secondary outcomes at study entry and completion are summarized in Table 5. Each variable was tested for change with a Wilcoxon signed-rank test, and no significant changes in any of the variables were observed.

To test for which characteristics were associated with better adherence, mixed-effects linear regression models were used to test for changes in adherence from baseline to study endpoints for total, visit, dietary log, and exercise point adherence, and this is summarized in Table 6. Patients in the Gain Group had a significant association for higher total, dietary log, and step goal adherence, but not clinic visits. Private insurance was associated with higher adherence for all adherence categories. Table 7 summarizes the medications associated with weight loss or gain that were prescribed to the study patients before or during the study. Of note, the number of patients on medications was not significantly different in either study group. In all, 33 adolescents (55%) completed their 6-month visit, with 9 (47%) in the CG, 10 in the LG (48%), and 14 in the GG (70%), *p* = 0.25 as by Fisher’s exact test. 

### 3.2. Economic Analysis

**Figure 6:**
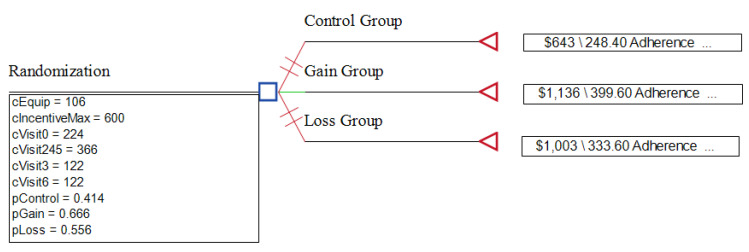
TreeAge derived decision tree cost-effective analysis.

Using TreeAge Pro Version 2023 software (Copyright 1988–2023 TreeAge Software, LLC, Williamstown, MA, USA) a decision tree was constructed for each study arm, as depicted in Figure 6. Assumptions on cost are described in Table 8. The GG arm proved more cost-effective than the LG arm, with an incremental cost-effective ratio (ICER) of 3.26 compared to USD 4.23 per incentive point, with the Control Group as a reference. Three-way sensitivity analysis was carried out, varying the adherence probability of each group by 10%. The GG had a more favorable ICER than the LG in all variations tested. 

## 4. Discussion

Our study demonstrates that a financial incentive in the form of a rechargeable gift card will increase adherence to a pediatric obesity treatment intervention. This is consistent with previous studies relating to other health conditions and adults [15,23,24,26,27]. Similar to other studies, our results suggest that the effect diminishes with time. This effect was described by Tulsky et al. in a study of financial incentives with prophylactic tuberculosis treatment in adults challenged by homelessness, in which adherence dropped from 100% to under 60% over six months [16]. We saw a similar effect in a study using small prizes to improve healthier food selection in elementary school students, with a 50% drop in healthier food selection in less than a month [15]. With this study, we show that incentives that are gain-framed appear to be more effective than loss-framed incentives. These results run counter to Behavioral Economic Prospect Theory, which suggests that the strong impulse of loss aversion will lead to loss-framed incentives being superior to gain-framed ones [38]. The results are consistent, however, with those reported by Malik et al., whose study of 34 children showed a small advantage of gain-framed compared to loss-framed incentives (45% versus 43%) in terms of improving acceptable “time in range” for adolescents’ self-monitoring with type 1 diabetes [39].

It is interesting to note that although we predicted that loss-framed incentives would be more effective than gain-framed based on Prospect Theory, in a prior survey of patients in our program, 53% of youths and 68% of their caretakers predicted that a gain-framed incentive would be superior [31]. These findings seem to make sense when considering human development stages. It has been noted that loss aversion seems to increase with age in adults [40]. Additionally, adolescents may not have the level of abstract thinking necessary to appreciate the complexity of a virtual account and losing money from it [41]. Our study also shows that gain-framed incentives are more cost-effective than loss-framed incentives. Of note, our linear regression results confirm that the gain incentives are associated with greater adherence. The linear regression also suggests that those with private insurance were more likely to have higher adherence scores than those with state-funded insurance, a maker of lower socioeconomic status, where the reward might be predicted to have a greater impact. This finding implies that other stressors associated with lower economic status might affect adherence. 

Given that adolescents with obesity are at risk for developing type 2 diabetes, cardiovascular disease, metabolic dysfunction-associated steatotic liver disease, and other chronic conditions, the AAP recommends intensive lifestyle management and monitoring metabolic parameters, such as liver enzymes, lipid profile, and percent Hemoglobin A1c in adolescents with obesity [7]. In a systemic review of lifestyle interventions for children and adolescents with obesity, Ho et al. demonstrated improvements in BMI, lipid profile, and insulin levels [42]. Although none of the secondary outcomes achieved significant changes, we noted a numerical improvement in BMI status in all three groups and in HgbA1c in the GG and LG. The ALT numerically, but non-significantly, improved in the GG and CG. Veggie meter scores have been used as a non-invasive, non-objective method of measuring skin carotenoid levels and as a proxy for assessing fruit and vegetable consumption in children and adolescents [43,44]. We saw small, nonsignificant (less than 10%) increases in the GG and CG. While these small improvements may be suggestive of clinical, lifestyle, and metabolic improvement, they should be interpreted with caution as our number of patients is small. 

Several of our secondary findings correlate with the literature. Multiple studies have identified the necessity of engaging parents and families in obesity prevention interventions as they play a dominant role in the lives of children [7,8,12,45]. We too find that, in our intervention, the measured activity with the greatest potential role for parental involvement, clinic visits, three in-person and four via telehealth, were the only measured activity that was not significantly different across arms of the trial (see Table 3).

In addition, our sample of children with obesity comprised 67% white children and 33% nonwhite (25% African American and 8.3% multi-racial (N = 5)). Although we did not analyze our differences in means by race, we saw very little evidence of a strong racial effect in our regression analyses. Whites were not statistically significantly different from African Americans on any measure except clinic visits. Multi-racial children (albeit with a very small number of observations) were statistically significantly more likely to achieve higher adherence points and to respond to their dietary survey than African American participants. However, it has been noted that African American children are more likely to drop out of treatment protocols and that the socioeconomic and cultural contexts across diverse communities require particular attention. For example, Alkhatib and Obita found that obesity intervention programs work better in minority communities when parents and community stakeholders co-design the programs [45]. This is in part because different communities respond differently to interventions—with Hispanic children responding well to educational interventions and Chinese Americans responding well to online and computer-based interventions; however, neither of these types of interventions is as well received or effective among African American communities. 

The results of the linear regression further suggest that those with private insurance were more likely to have higher adherence scores than those with state-funded insurance, a maker of lower socioeconomic status, where the reward might be predicted to have a greater impact. This finding implies other stressors associated with lower economic status, such as fear of victimization by social services, cultural and religious traditions, and racial stigma related to child weight in the wider community, might be affecting adherence [46].

Lastly, our cost-effectiveness analysis shows that gain-framed incentives are more cost-effective than loss-framed ones.

## 5. Limitations

Our study, of course, has several limitations. Our numbers are relatively small and were based on a sample size to test only for the primary outcome of total adherence points. This limits the conclusions that can be made on the clinical effectiveness of improving secondary outcomes, such as BMI status (%BMIp95), metabolic laboratory parameters, and obesity-related co-morbidities, as well as describing the effects of ethnic/social disparities on response. Given that most of the parameters moved in a favorable albeit statistically insignificant manner, it is likely that with a larger definitive study, there will be an improvement in BMI and metabolic status. Additionally, the length of this study was only 6 months. It remains to be seen whether the effects are maintained with and without incentives. Our results show that the effect on adherence, like other studies, decreases with time. Moreover, we were not able to explore the association of menarche directly on adherence and secondary endpoints. We can, however, infer that menarche does not seem to affect adherence, as sex was not associated with adherence in the linear regression. Another shortcoming of our study is that we depended on the randomization technique to ensure that the characteristics of the three groups were similar. Despite this limitation, however, the characteristics of the three groups, as described in Table 2, were remarkably similar in terms of demographic and clinical characteristics. We only wanted objective, verifiable data for adherence; we do not know whether dietary intake changed during this study and, ultimately, our study relates to behavioral interventions designed to augment the current standard of care. We did, however, include the veggie meter score as a secondary outcome as it is an objective measure. While not significant, there was a numerical increase in the CG and GG. Finally, because of the nature of the intervention, our study was not blinded; thus, patient and observer bias may have influenced our results. 

## 6. Conclusions

It appears that incentives, in general, work in the pediatric obesity treatment setting for improving adherence and that gain-framed incentives specifically are superior to loss-framed incentives. The potential use of incentives with pediatric obesity treatment should be considered within the context that the causes of obesity in adolescents are enormously complex, and factors beyond nutrition, physical activity, and obesity treatment program visits contribute and may need to be addressed. Specifically, as described by Alkhatib and Obita, the cultural context and presence of socioeconomic barriers need to be considered and incorporated into pediatric obesity interventions to achieve greater adherence and, as a result, healthier lives for all children [46,47].

## Figures and Tables

**Table 1:** Adherence index/incentive scheme.

Type of Activity		When Available	Value Representing Adherence	Adherence Index Points	Maximum Points per Month	Maximum Incentive per Month
Study Participation		Monthly	Yes	10 points per month	10 points	USD 10
Exercise Adherence	Daily steps by tracker	Daily	0 to 3000 daily steps	0 points per day	30 points	USD 30
			3001 to 5000 daily steps	0.5 points per day		
			5001 or more daily steps	1 point per day		
Dietary Adherence	Completing goal log answering questions: 1. Did you eat breakfast yesterday? y/n (meal eaten before 10:30 a.m.) 2. How many fruits or vegetables eaten yesterday? 0 to >10 3. How many sugary drinks did you drink yesterday? 0 to >10 4. Did you eat after 7 p.m. yesterday? y/n	Daily	Daily survey completed	1 per day	30 points	USD 30
Clinical Adherence	In clinic (0, 3, 6 mos.) or telehealth (1, 2, 4, 5 mos.)	Monthly	Monthly visit attended	30 points per month	30 points	USD 30
Maximum Total					100 points	USD 100

**Table 2:** Baseline unadjusted characteristics of study participants.

Characteristic	Overall, N = 60 ^1^	Control, N = 19 ^1^	Loss, N = 21 ^1^	Gain, N = 20 ^1^	*p*-Value ^2^
Age	15.2 (1.4)	15.2 (1.3)	15.4 (1.4)	15.2 (1.4)	0.857
Sex					0.901
Male	23 (38%)	8 (42%)	8 (38%)	7 (35%)	
Female	37 (62%)	11 (58%)	13 (62%)	13 (65%)	
Race					0.908
African American	15 (25%)	5 (26%)	6 (29%)	4 (20%)	
Caucasian	40 (67%)	12 (63%)	13 (62%)	15 (75%)	
Multi-racial	5 (8.3%)	2 (11%)	2 (9.5%)	1 (5.0%)	
Ethnicity					0.861
Hispanic	5 (8.3%)	2 (11%)	2 (9.5%)	1 (5.0%)	
Non-Hispanic	55 (92%)	17 (89%)	19 (90%)	19 (95%)	
Income					0.445
Less than USD 24,999	2 (3.9%)	0 (0%)	2 (11%)	0 (0%)	
USD 25,000–USD 49,999	16 (31%)	5 (33%)	7 (39%)	4 (22%)	
USD 50,000–USD 74,999	8 (16%)	2 (13%)	1 (5.6%)	5 (28%)	
USD 75,000–USD 99,999	8 (16%)	1 (6.7%)	3 (17%)	4 (22%)	
USD 100,000–USD 149,999	10 (20%)	4 (27%)	2 (11%)	4 (22%)	
>USD 150,000	7 (14%)	3 (20%)	3 (17%)	1 (5.6%)	
Insurance type					0.708
Public (Medicaid)	25 (46%)	7 (41%)	8 (42%)	10 (56%)	
Private	28 (52%)	10 (59%)	10 (53%)	8 (44%)	
Military	1 (1.9%)	0 (0%)	1 (5.3%)	0 (0%)	
%BMIp95	140.2 (23.5)	137.7 (18.7)	142.0 (25.6)	140.7 (26.2)	0.971
HgA1C%	5.5 (0.3)	5.5 (0.3)	5.5 (0.3)	5.5 (0.4)	0.853
Total cholesterol mg/dL	156.0 (22.2)	159.2 (23.6)	152.1 (23.4)	156.8 (20.9)	0.694
HDL mg/dL	41.0 (6.8)	41.5 (5.4)	40.7 (9.1)	40.9 (6.0)	0.552
LDL mg/dL	95.4 (22.3)	96.7 (22.7)	90.9 (18.6)	98.2 (25.2)	0.685
Triglycerides mg/dL	106.3 (59.5)	105.9 (41.3)	103.4 (36.8)	108.9 (84.5)	0.596
AST U/L	24.4 (9.6)	23.5 (7.1)	23.7 (10.5)	25.6 (10.9)	0.755
ALT U/L	30.3 (23.7)	25.2 (12.7)	28.7 (18.5)	35.6 (32.6)	0.953
Systolic BP mm	117.1 (9.0)	116.3 (8.5)	116.5 (9.5)	118.6 (9.2)	0.774
Diastolic BP mm	67.7 (8.8)	66.7 (6.0)	67.6 (9.5)	68.9 (10.6)	0.840
Veggie Meter	179.8 (64.6)	146.5 (60.1)	205.5 (72.1)	182.7 (47.5)	0.021

^1^ Mean (SD); n (%); ^2^ Kruskal–Wallis rank sum test; Pearson’s Chi-squared test; Fisher’s exact test; Abbreviations: High-Density Lipoprotein = HDL; Low-Density Lipoprotein = LDL; Hemoglobin A1C percent = HgA1C%; Alanine Aminotransferase = ALT; and Aspartate Aminotransferase = AST.

**Table 3:** Adherence points by group per month.

Characteristic (Points Per Month)	Overall N = 60 ^1^	Control N = 19 ^1^	Loss N = 21 ^1^	Gain N = 20 ^1^	*p*-Value ^2^
Exercise (step goal)	9.6 (9.9)	3.9 (6.5)	10.0 (9.8)	14.6 (10.2)	0.003
Dietary	13.4 (10.7)	8.1 (8.9)	13.9 (11.5)	17.9 (9.6)	0.016
Clinic visits	20.7 (9.3)	18.7 (10.7)	19.9 (9.6)	23.5 (6.9)	0.386
Total points	54.1 (25.7)	40.6 (21.3)	54.9 (25.9)	66.0 (24.1)	0.011

^1^ Mean (SD); ^2^ Kruskal–Wallis rank sum test.

**Table 4:** Fitbit wear time and steps per day by group.

Characteristic	Overall N = 60 ^1^	Control N = 19 ^1^	Loss N = 21 ^1^	Gain N = 20 ^1^	*p*-Value ^2^
Fitbit-days worn	88.5 (72.9)	45.4 (63.4)	94.1 (69.3)	118.4 (70.2)	0.010
Mean steps per day	6500.7 (3166.7)	5369.5 (3562.3)	6762.0 (3097.4)	7155.7 (2815.5)	0.332

^1^ Mean (SD); ^2^ Kruskal–Wallis rank sum test.

**Table 5:** (1) Pre- and post-comparisons of characteristics in the Control Group. (2) Pre- and post-comparisons of characteristics in the Loss Group. (3) Pre- and post-comparisons of characteristics in the Gain Group.

**(1)**			
	**Pre vs. Post in Control**		
**Characteristic**	**Pre, N = 19 ^1^**	**Post, N = 19 ^1^**	***p*** **-Value ^2^**
%BMIp95	138 (19)	135 (9)	>0.999
Systolic bp mm	116 (9)	116 (12)	0.623
Diastolic bp mm	67 (6)	69 (9)	0.858
Veggie meter score	146 (60)	151 (25)	0.447
HgA1C %	5.46 (0.28)	5.46 (0.24)	0.832
HDL mg/dL	42 (5)	43 (8)	0.469
LDL mg/dL	97 (23)	101 (25)	>0.999
Cholesterol mg/dL	159 (24)	166 (29)	0.578
Triglycerides mg/dL	106 (41)	106 (30)	0.309
AST U/L	24 (7)	20 (9)	0.233
ALT U/L	25 (13)	21 (13)	0.787
**(2)**			
	**Pre vs. Post in Loss**		
**Characteristic**	**Pre, N = 21 ^1^**	**Post, N = 21 ^1^**	***p*** **-Value ^2^**
%BMIp95	142 (26)	132 (13)	>0.999
Systolic bp mm	117 (9)	115 (8)	0.437
Diastolic bp mm	68 (10)	69 (5)	0.394
Veggie meter score	206 (72)	190 (53)	0.933
HgA1C %	5.52 (0.26)	5.45 (0.29)	>0.999
HDL mg/dL	41 (9)	42 (6)	>0.999
LDL mg/dL	91 (19)	90 (37)	0.423
Cholesterol mg/dL	152 (23)	157 (46)	0.423
Triglycerides mg/dL	103 (37)	127 (44)	0.181
AST U/L	24 (10)	22 (8)	0.423
ALT U/L	29 (18)	29 (17)	0.789
**(3)**			
	**Pre vs. Post in Gain**		
**Characteristic**	**Pre, N = 20 ^1^**	**Post, N = 20 ^1^**	***p*** **-Value ^2^**
%BMIp95	142 (26)	132 (13)	>0.999
Systolic bp mm	119 (9)	120 (11)	0.637
Diastolic bp mm	69 (11)	70 (8)	0.972
Veggie meter score	183 (47)	198 (51)	0.675
HgA1C %	5.49 (0.42)	5.41 (0.45)	0.855
HDL mg/dL	40.9 (6.0)	38.9 (7.8)	0.684
LDL mg/dL	98 (25)	105 (17)	0.402
Cholesterol mg/dL	157 (21)	164 (19)	0.498
Triglycerides mg/dL	109 (84)	134 (121)	>0.999
AST U/L	26 (11)	22 (10)	0.075
ALT U/L	36 (33)	31 (29)	0.271

^1^ Mean (SD); ^2^ Wilcoxon signed rank test; Abbreviations: High-Density Lipoprotein = HDL; Low-Density Lipoprotein = LDL; Hemoglobin A1C percent = HgA1C%; Alanine Aminotransferase = ALT; and Aspartate Aminotransferase = AST.

**Table 6:** Summary of mixed-effects linear regression models in which adherence points (total, visit, dietary log, and exercise, as determined by steps) are the dependent variables. Reference of each characteristic appears as (—).

Characteristic	Total			Visit			Diet			Exercise		
	Beta	95% CI ^1^	*p*-Value	Beta	95% CI ^1^	*p*-Value	Beta	95% CI ^1^	*p*-Value	Beta	95% CI ^1^	*p*-Value
Study Group												
Control	—	—		—	—		—	—		—	—	
Loss	13	−1.3, 27	0.074	0.59	−5.0, 6.2	0.833	4.7	−1.1, 10	0.111	6.5	0.43, 13	0.036
Gain	**28**	**13, 42**	**<0.001**	5.3	−0.40, 11	0.067	**10**	**4.6, 16**	**<0.001**	**12**	**5.9, 18**	**<0.001**
Age	1.6	−3.0, 6.2	0.483	1.1	−0.65, 2.9	0.204	0.00	−1.9, 1.9	0.999	0.93	−1.0, 2.9	0.341
Sex												
Male	—	—		—	—		—	—		—	—	
Female	−2.8	−15, 9.6	0.650	−1.7	−6.5, 3.2	0.492	1.6	−3.4, 6.6	0.518	−1.6	−6.8, 3.7	0.545
Insurance type												
Public (Medicaid)	—	—		—	—		—	—		—	—	
Private	**19**	**6.1, 32**	**0.005**	**5.5**	**0.50, 10**	**0.032**	**8.3**	**3.2, 13**	**0.002**	**6.3**	**0.93, 12**	**0.022**
Military	38	−7.9, 83	0.103	11	−6.4, 29	0.204	23	4.7, 41	0.015	3.7	−15, 23	0.693
Race												
African American	—	—		—	—		—	—		—	—	
Caucasian	13	−2.4, 28	0.096	8.1	2.2, 14	0.009	2.9	−3.2, 9.0	0.345	1.3	−5.1, 7.7	0.679
Multi-racial	26	2.7, 50	0.030	6.5	−2.7, 16	0.159	9.7	0.35, 19	0.042	9.1	−0.70, 19	0.068
Ethnicity												
Hispanic	—	—		—	—		—	—		—	—	
Non-Hispanic	−11	−32, 10	0.303	−0.96	−9.1, 7.2	0.813	−3.7	−13, 5.5	0.420	−1.0	−11, 8.6	0.831
p95%tile-BMI ^2^	0.22	−0.04, 0.47	0.094	0.02	−0.08, 0.12	0.638	**0.11**	**0.01, 0.21**	**0.034**	0.07	−0.04, 0.18	0.188

^1^ CI = Confidence Interval; ^2^ p95%tile-BMI= percent of the 95th percentile for age and sex.

**Table 7:** Summary of patient medications associated with weight gain or loss by study group.

Medication or Class	Control, N = 19	Loss, N = 21	Gain, N = 20	*p* ^2^
Metformin	3 (16%)	2 (10%)	3 (15%)	0.802
GLP1-RA ^1^	1 (5%)	1 (5%)	1 (5%)	1.000
Stimulant	0 (0%)	3 (14%)	0 (0%)	0.100
Bupropion	0 (0%)	1 (5%)	0 (0%)	1.00
Topiramate	0 (0%)	1(5%)	0 (0%)	1.00
Duloxetine	0 (0%)	1 (5%)	0 (0%)	1.00
Total Patients receiving medications	4 (21%)	7 (33%)	4 (20%)	0.407

^1^ GLP1-RA = Glucagon-lie peptide-1 receptor agonist (Liraglutide or Semaglutide); ^2^ Fisher’s exact test.

**Table 8:** Study costs.

Item	Cost	Comments
Equipment		
Fitbit	USD 66	
Scale	USD 40	
Provider Visits *		In the decision tree analysis, visits 2, 4, and 5 were bundled together for a total cost of USD 366 as the probability of keeping the visits are equal
Initial visit MD	USD 153	
Initial visit dietitian	USD 37	
Initial visit exercise physiologist	USD 34	
Total cost of visit 1	USD 224	
Follow-up MD visit	USD 51	
Follow-up dietitian visit	USD 37	
Follow-up exercise physiologist	USD 34	
Total follow-up visit	USD 122	
Cost of visits 2, 4, and 5	USD 366	
Maximum incentive study incentive Study participation incentive (Control group Only)	USD 600 USD 60	The actual incentive can vary depending on the level of adherence, as outlined in Table 1.

* From Medicare costs given on Clearhealthcosts.com (accessed on 29 September 2024) [37].

## Data Availability

The original contributions presented in the study are included in the article, further inquiries can be directed to the corresponding author.
